# Sodium Houttuyfonate Alleviates Post-infarct Remodeling in Rats via AMP-Activated Protein Kinase Pathway

**DOI:** 10.3389/fphar.2018.01092

**Published:** 2018-09-27

**Authors:** Cheng Zheng, Jia-Feng Lin, Zhong-Hao Lin, Wei-Qian Lin, Saroj Thapa, Yuan-Zheng Lin, Hao Lian, Zhi-Rui Liu, Jia-Hui Chen, Xiao-Wei Li

**Affiliations:** ^1^Department of Cardiology, The Second Affiliated Hospital and Yuying Children’s Hospital of Wenzhou Medical University, Wenzhou, China; ^2^Department of Cardiology, Taishun General Hospital, Wenzhou, China

**Keywords:** Sodium Houttuyfonate, myocardial infarction, post-infarct remodeling, AMP-activated protein kinase, cardiac inflammatory response, cardiac fibrotic response

## Abstract

With the chronic ischemia persisting after acute myocardial infarction, the accompanying low-degree inflammation and subsequent fibrosis result in progression of cardiac remodeling and heart failure. Recently, Sodium Houttuyfonate (SH), a pure compound extracted from *Houttuynia cordata*, has been confirmed exerting anti-inflammatory and anti-fibrotic effects under diseased situations. Here, we aimed to investigate whether SH could reverse the cardiac remodeling post-myocardial infarction by alleviating cardiac inflammation and fibrosis. Left anterior descending coronary artery of adult male Sprague-Dawley rats was ligated to elicit myocardial infarction. Low and high dose of SH was administered by oral gavage for four consecutive weeks post-myocardial infarction. Long-term SH treatment decreased heart rate, heart weight/ body weight (HW/BW), and left ventricle weight/body weight (LVW/BW), reduced cardiac expression of brain natriuretic peptide (BNP), improved left ventricular heart function, and ameliorated the histopathological changes caused by myocardial infarction. Western blotting revealed the expression of tumor necrosis factor-α (TNF-α), interleukin-6 (IL-6), transforming growth factor-β (TGF-β), collagen I, and collagen III of the infarcted ventricle were reduced by SH treatment. Meanwhile, we found that SH treatment post-myocardial infarction activated AMP-activated protein kinase (AMPK) and suppressed nuclear factor-κB p65 (NF-κB p65). Furthermore, on H9C2 cells induced hypoxic injury with cobalt chloride (CoCl_2_), the reduction of inflammatory cytokines (IL-6, TNF-α, and TGF-β), activation of AMPK, and suppression of NF-κB p65 were also observed by SH treatment. However, transfection of H9C2 with AMPKα siRNA blunted the suppression of NF-κB p65 and inflammatory cytokines (IL-6, TNF-α, and TGF-β) by SH post-hypoxia. Taken together, these findings suggested that long-term administration of SH post-myocardial infarction reduced cardiac inflammatory and fibrotic responses, and reversed cardiac remodeling process. The underlying mechanism may be activating AMPK and suppressing NF-κB pathway.

## Introduction

The process of cardiac remodeling post-myocardial infarction initiated immediately after acute myocardial infarction and was defined as maladaptive responses in both infarcted and non-infarcted regions of the ventricle, leading to impaired contractile function, ventricular dilatation, and heart failure. Recent studies have revealed that the persisting low-degree inflammatory and fibrotic responses participated in the adverse cardiac remodeling process post-myocardial infarction and speeded up the development of chronic heart failure (Li et al., 2006; Takahashi et al., 2008; Hamid et al., 2011; Sobirin et al., 2012; Shivshankar et al., 2014; Wang B. et al., 2017). Thus, modulation of the inflammatory and fibrotic responses may be a promising therapy for adverse cardiac remodeling and heart failure post-myocardial infarction.

Sodium Houttuyfonate (SH), which was a pure compound extracted from a Chinese herb *Houttuynia cordata*, has shown potent anti-inflammatory and anti-fibrotic activities on various animal and cell models of diseases, like lipopolysaccharide (LPS)-induced acute lung injury (ALI), LPS-induced bovine endometrial epithelial cell inflammation, smoking-induced chronic obstructive pulmonary disease (COPD), ventricular remodeling induced by abdominal aortic banding and so on (Gao et al., 2010; Du et al., 2012; Kang and Koppula, 2014a, 2015; Xu et al., 2015; Zhu et al., 2015; Wu et al., 2017). Based on these findings, we doubted whether SH with an inherent anti-inflammatory and anti-fibrotic potency, could ameliorate post-infarct cardiac remodeling and heart failure.

Adenosine monophosphate-activated protein kinase (AMPK), which consisted of three subunits, α, β, γ, that together made a functional enzyme, worked as an energy sensor to provide metabolic adaptations under the ATP-deprived conditions, such as ischemia and nutritional stress (Calvert et al., 2008; Quan et al., 2018). Activation of AMPK has been confirmed exerting pleiotropic functions against inflammatory response and tissue fibrosis and owning beneficial properties in a series of diseases (Peairs et al., 2009; Chiang et al., 2017; Kim et al., 2017). Intriguingly, recent studies have found activation of AMPK could be elicited or enhanced by *H. cordata* treatment in diseased situations (Peairs et al., 2009; Xu et al., 2015; Wang J.H. et al., 2017).

In this article, we mainly focused on investigating two unknown entities, whether SH treatment alleviated the cardiac remodeling post-myocardial infarction, and if so, whether AMPK activation was involved in the benefit of SH treatment.

## Materials and Methods

**FIGURE 1:**
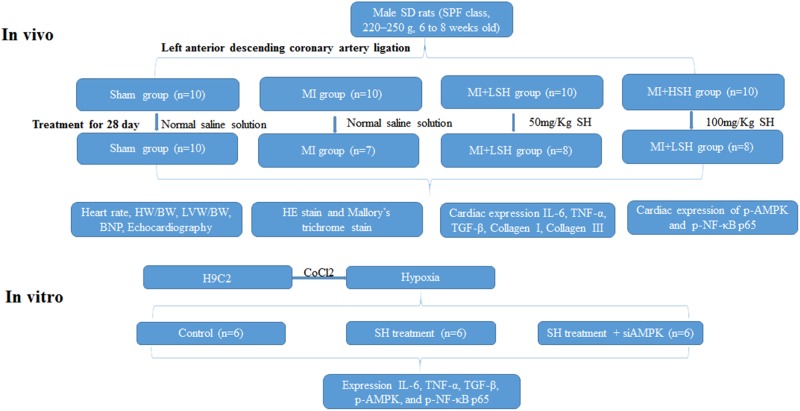
Experimental design. Sham represented sham operation group; MI represented myocardial infarction group; MI+LSH represented myocardial infarction group with low dose Sodium Houttuyfonate (SH) treatment; MI+HSH represented myocardial infarction group with high dose treatment.

The experimental design of our study was presented in **Figure 1**.

### Reagents

Sodium Houttuyfonate, Hematoxylin and Eosin (HE) stain kit and Mallory’s trichrome stain kit were purchased from Solarbio Science & Technology (Shanghai, China). The antibodies phospho-AMPKα (Thr172)(40H9)Rabbit mAb#2535, AMPKα(D63G4) Rabbit mAb#5832, NF-κB p65 (D14E12) XP^®^ Rabbit mAb #8242, Phospho-NF-κB p65 (Ser536) (93H1) Rabbit mAb #3033 were purchased from Cell Signaling Technology (Danvers, MA, United States). The antibodies β-Actin (4D3) monoclonal mouse antibody# BS6007M was purchased from Bioworld Technology (Nanjing, China). The antibodies IL-6 mouse Antibody#53324 was purchased from R&D System Technology (Minneapolis, MN, United States). The antibodies tumor necrosis factor (TNF)-α#Rabbit ab6671, Rabbit polyclonal to TGFβ1#ab92486, Rabbit polyclonal to Collagen I#ab34710, and Rabbit polyclonal to Collagen III#ab7778 and Rabbit monoclonal [EPR16891] to GAPDH, anti-BNP antibody ab19645 were purchased from Abcam (Shanghai, China). Horseradish peroxidase (HRP) conjugated goat anti-rabbit IgG antibodies and anti-mouse IgG antibodies were purchased from Biosharp (Hefei, China). Cell counting kit-8 was purchased from Dojindo Molecular Technologies (Rockville, MD, United States). SiAMPKα was bought from Riobio technologies (Guangzhou, China). Lipofectamine^®^ 2000 reagent was bought from Invitrogen (Carlsbad, CA, United States).

### Animal Preparation

A total of 80 Male Sprague-Dawley rats (specific pathogen-free (SPF) class, 220–250 g, 6 to 8 weeks old) were purchased from Shanghai Laboratory Animal Center of China (Shanghai SLAC Laboratory Animal Co., Ltd.) and were kept under the SPF conditions (24 ± 1°C, 45 ± 10% humidity) with a 12-h light/dark cycle daily and food and water available *ad libitum* in the Wenzhou Medical University animal facilities. All animal experiments were approved by the Animal Ethics Committee of Wenzhou Medical University (number wydw2014-0058) and conformed to the Guide for the Care and Use of Laboratory Animals by the National Institutes of Health.

### Myocardial Infarction and Animal Model Establishment

After induction of anesthesia (urethane 1.5 g/kg, administered intraperitoneally), the rats received left parasternotomy under transtracheal ventilation. After the hearts were deprived of pericardium and fully exposed, the left anterior descending coronary artery was visualized directly and ligated by a 6-0 Prolene suture at about 2 mm from its origin. All the operations adhered strictly to the aseptic techniques. Ten rats were randomly selected and received sham operation. All the other rats received the coronary artery ligation to induce myocardial infarction, the surviving rats after operation were then assigned blindly to three groups. The following treatments were given, respectively: sham-operated rats with normal saline solution administered by oral gavage once daily for 28 days (Sham, *n* = 10), myocardial infarction rats with normal saline solution administered by oral gavage once daily for 28 days (MI, *n* = 10), myocardial infarction rats with low dose SH of 50 mg/Kg administered by oral gavage once daily for 28 days (MI+LSH, *n* = 10), myocardial infarction rat with high dose SH of 100 mg/Kg administered by oral gavage once daily for 28 days (MI+HSH, *n* = 10). On the 28th day, all the surviving rats from each group were sacrificed and their hearts were obtained for histopathological and biochemical examinations. All rats were anesthetized (urethane 1.5 g/kg, administered intraperitoneally) prior to sacrifice. Until sufficient sedation was achieved, the chest of rats were opened and the hearts were obtained.

### Doppler Echocardiography Study

Transthoracic echocardiography was performed on the 28th day after ligation with vevo1100 cardiovascular research ultrasound machine (Visualsonics, Japan). At the papillary muscle level, the LV end-diastolic diameter (LVEDd) and LV end-systolic diameter (LVESd) were measured by short-axis views of M-mode tracings from the anterior to posterior LV wall, the left ventricular ejection fraction (LVEF) was evaluated by the Simpson approach. All measurements were performed by an experienced technician who was blinded to the study groups.

### Heart Rate Calculation

Prior to sacrifice, the heart rate of rats was recorded for 10 min by a computer-based electrical physiology system (PowerLab 8/36; AD Instruments, Colorado Springs, CO, United States).

### HW/BW and LVW/BW Calculation

The heart weight (HW), left ventricle weight (LVW), body weight (BW), and the ratio of HW and LVW to BW (HW/BW and LVW/BW) were calculated on the 28th day.

### Hematoxylin and Eosin Stain and Mallory’s Trichrome Stain

The left ventricles of rats were obtained on the 28th day after ligation. LV middle ring (the middle 1/3 of the left ventricle) was embedded in paraffin, sectioned into 5-um-thick slices and treated with HE stain and Mallory’s trichrome stain, respectively. The infiltration of inflammatory cells and fibrotic scar tissue were observed at infarct zone, peri-infarct border zone (within 0.5–2 mm from infarct zone) and remote zone (within 2–3 mm from infarct zone).

### Western Blotting

The proteins were extracted from the left ventricle and H9C2 cells. Each sample was separated by SDS–PAGE and transferred to a polyvinylidene difluoride membrane. The membrane was blocked in 5% non-fat dry milk. After being incubated with the specific primary antibody (anti-phospho-AMPKα, anti-AMPKα, anti-NF-κB p65, anti-phospho-NF-κB p65, anti-IL-6, anti-TNF-α, anti-TGFβ, anti-collagen I, anti-collagen III, anti-β-actin, anti-GAPDH) and secondary antibody (HRP conjugated goat anti-rabbit and anti-mouse antibodies), the membrane was detected with the enhanced chemiluminescence detection system (Millipore, Billerica, MA, United States).

### Cellular Experiments

The rat cardiomyoblast cell line, H9C2, was obtained from the Cell Bank of the Chinese Academy of Sciences (Shanghai, China) and cultured in Dulbecco’s modified Eagle’s medium containing 10% fetal bovine serum. The cells were maintained at 37°C in a humidified atmosphere with 5% CO_2_. H9C2 cells (100 μl/well) were seeded into 96-well plate at a density of 3.0 × 10^4^ cells/ml and incubated for 24 h in medium containing different concentrations (0, 200, 400, 600, 800, 1000, 1200, and 14000 μM) of Cobalt chloride (CoCl_2_). Cell viability was tested with cell counting kit-8, the concentration of CoCl_2_ resulting in half reduction of the cell viability was chosen to induce chemical hypoxia_._ In addition, H9C2 cells were seeded into 96-well plate at a density of 3.0 × 10^4^ cells/ml and incubated for 24 h in medium in the presence of different concentrations (0, 10, 20, 30, 40, 50, 60, 70, and 80 μg/ml) of SH. Cell viability was also evaluated with CCK-8, the concentrations of SH resulting in a reduction of the cell viability was excluded. In order to verify the most appropriate concentration of SH for H9C2 cells post-hypoxia, the H9C2 were induced hypoxia first and then treated with different concentrations of SH, which were in the range of no cell viability reduction. Changes in protein expression were measured by western blotting.

Moreover, to verify whether the effect of SH on H9C2 cells post chemical hypoxia was related to AMPK, siRNA-mediated knockdown of AMPKα was performed. H9C2 cells were transfected with siAMPKα by using Lipofectamine^®^ 2000 reagent for 6 h at 37°C, then treated with CoCl_2_ for 24 h and incubated with fresh cell culture medium containing the most appropriate SH for the next 24 h. Changes in protein expression were measured by western blotting.

### Statistical Methods

Statistical analyses were performed using SPSS 14 software (Unicom, Mission Hills, CA, United States). Data were expressed as mean ± standard deviation. Shapiro–Wilk test and Kolmogorov–Smirnov test were used for normal distribution. If distribution of the data is normal, outcomes are compared among groups using 1-way ANOVA followed by the Dunnett multiple-comparison test. If not, Wilcoxon’s rank sum test should be applied instead. A value of *p* < 0.05 was considered significant.

## Results

### Sodium Houttuyfonate Treatment Did Not Improve the Survival of Rats Post-myocardial Infarction, However, It Reduced Heart Rate, HW/BW and LVW/BW

In this study, 10 rats received sham operation and were included in the Sham group. The other 70 rats were all referred to the procedure of left anterior descending coronary artery ligation, and only 37 rats survived the operation. Thirty rats were randomly selected from the 37 surviving rats and assigned blindly to three groups (MI, MI+LSH, MI+HSH groups) with 10 rats in each group.

We first investigated the effect of SH treatment in terms of survival analysis during the 28-day follow-up. In MI, MI+LSH and MI+HSH group, 7 rat deaths occurred within the first 3 days after myocardial infarction, 5 rats were supposed died of heart failure with the presence of edematous lungs and 2 rats died from myocardial rupture. There was no obvious survival difference among MI, MI+LSH and MI+HSH groups (MI 70% vs. MI+LSH 80% vs. MI+HSH 80%, *p* > 0.05), with three deaths in MI group, two deaths in both MI+LSH and MI+HSH groups. No animal death was observed in Sham group.

We also investigated whether SH treatment influenced heart rate (HR), HW/BW and LVW/BW of rats on the 28th day post-myocardial infarction. Compared with Sham group, rats in the MI group presented an increased HR, HW/BW and LVW/BW (*p* < 0.05), while SH treatment decreased the HR, HW/BW, and LVW/BW of the infarcted rats (*p* < 0.05) and more reduction of these parameters was observed in MI+HSH group, see **Table 1**.

**Table 1:** Rat characteristics and cardiac function results from each group.

Variable	Sham (*n* = 10)	MI (*n* = 7)	MI+LSH (*n* = 8)	MI+HSH (*n* = 8)
**Rat characteristics**				
Body weight, g	297.2 ± 14.21	291.28 ± 18.48	306.62 ± 10.42	301 ± 16.14
Heart weight, mg	837.1 ± 70.2	1292.86 ± 62.11*	1134.75 ± 44.46^∗#^	1030.5 ± 73.96^∗#∧^
Heart weight/body weight, mg/g	2.83 ± 0.33	4.45 ± 0.21*	3.71 ± 0.22^∗#^	3.44 ± 0.36^∗#∧^
Left ventricular weight, mg	578.9 ± 40.54	917.14 ± 40.48*	846.25 ± 26.34^∗#^	730.5 ± 39.53^∗#∧^
Left ventricular weight/body weight, mg/g	1.95 ± 0.18	3.16 ± 0.23*	2.76 ± 0.14^∗#^	2.43 ± 0.09^∗#∧^
**Cardiac function**			
Heart rate, bpm	390.8 ± 10.65	466.86 + 20.01^∗^	430.12 ± 9.43^∗#^	412 ± 12.2^∗#∧^
LVEDd, mm	5.15 ± 0.55	7.94 ± 0.29*	7.50 ± 0.26^∗#^	7.10 ± 0.24^∗#∧^
LVESd, mm	2.81 ± 0.49	6.58 ± 0.19*	5.99 ± 0.28^∗#^	5.40 ± 0.39^∗#∧^
LVEF, %	83.52 ± 5.03	42.83 ± 5.02*	48.90 ± 4.37^∗#^	55.70 + 6.90^∗#∧^


*Sham, Sham group; MI, myocardial infarction group; MI+LSH, myocardial infarction+low dose Sodium Houttuyfonate group; MI+HSH, myocardial infarction+high dose Sodium Houttuyfonate group; ^∗^compared with Sham group, *p* < 0.05; ^#^compared with MI group, *p* < 0.05; ^∧^compared with MI+LSH group, *p* < 0.05.*

### Administration of Sodium Houttuyfonate Improved Left Ventricular Function, Decreased Left Ventricular Diameters and Cardiac Expression of Brain Natriuretic Peptide (BNP) of Rats Post-myocardial Infarction

**FIGURE 2:**
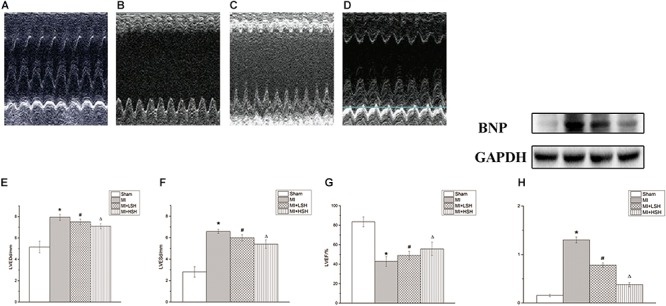
Sodium Houttuyfonate treatment alleviated the post-infarct remodeling. **(A–D)** Typical M-mode echocardiography at papillary muscle level of Rat from each group (**A**. Sham group, *n* = 10; **B**. MI group, *n* = 7; **C**. MI+LSH group, *n* = 8; MI+HSH, *n* = 8). **(E–G)** Comparison of echocardiographic parameters (LVEDd, LVEDd, LVEF) among four groups. **(H)** Comparison of BNP among four group. Data were expressed as means ± SD; LVEDd, left ventricular end-diastolic diameter; LVESd, left ventricular end-systolic diameter; LVEF, left ventricular ejection fraction; BNP, Brain natriuretic peptide. ^∗^Compared with Sham group, *p* < 0.05; ^#^compared with MI group, *p* < 0.05; ^Δ^compared with MI+LSH group, *p* < 0.05.

We further used three echocardiographic parameters, including LVEDd, LVESd, and LVEF, to evaluate the cardiac remodeling post-myocardial infarction. On the 28th day, we found that compared with rats from Sham group, there was an obvious adverse cardiac remodeling occurred on rats from MI group with enlarged left ventricle size and decreased left ventricular ejection function (*p* < 0.05). However, decreased left ventricular end-diastolic and end-systolic diameters with increased left ventricle ejection fraction was observed in SH treatment groups (*p* < 0.05). In addition, the improvement of echocardiographic parameters were more obvious in the MI+HSH group (*p* < 0.05), as shown in **Table 1** and **Figure 2**.

Brain natriuretic peptide (BNP) has been proved useful in the diagnosis of cardiac dysfunction and heart failure. Therefore, we also explored the effect of SH treatment on cardiac BNP level post-myocardial infarction. We found that compared with rats from Sham group, a remarkable elevation of cardiac BNP was observed in MI group (*p* < 0.05). SH treatment groups reduced the expression of BNP from the infarcted heart (*p* < 0.05). Additionally, the cardiac BNP were more reduced by high dose SH treatment (*p* < 0.05), see **Figure 2**.

### Administration of Sodium Houttuyfonate Alleviated the Infiltration of Inflammation and Fibrosis in the Left Ventricle Post-myocardial Infarction

**FIGURE 3:**
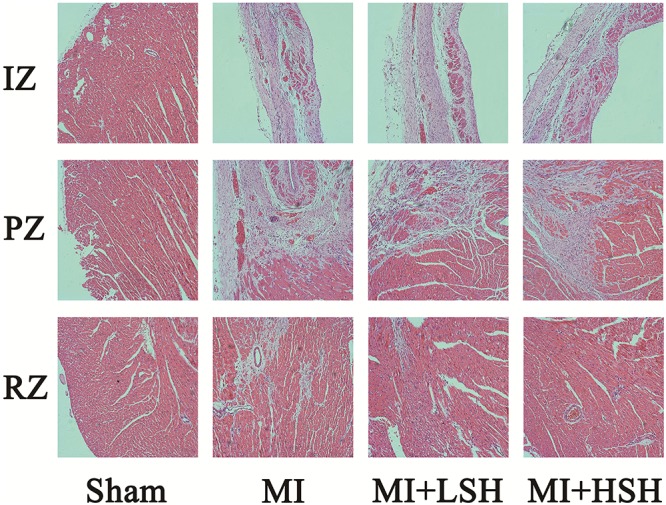
Hematoxylin and Eosin stain revealed that SH treatment ameliorated the inflammatory cell infiltration in infarcted zone, peri-infarcted zone and remote zone. IZ, infarcted zone; PZ, peri-infarcted zone; RZ, remote zone (magnification ×100).

**FIGURE 4:**
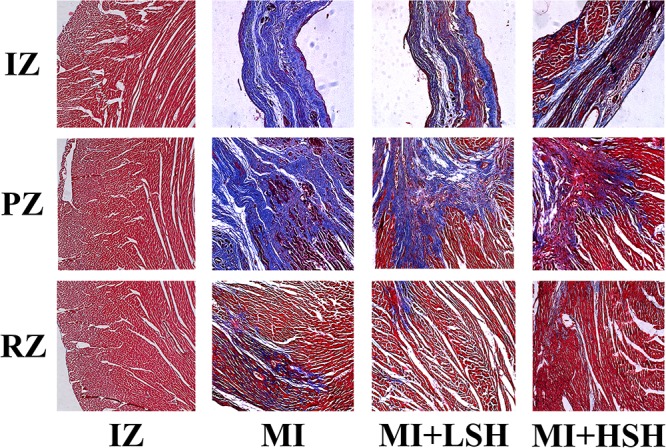
Mallory’s trichrome stain revealed that SH treatment ameliorated the fibrosis infiltration in infarcted zone, peri-infarcted zone, and remote zone. IZ, infarcted zone; PZ, peri-infarcted zone; RZ, remote zone (magnification ×100).

We next examined the effect of SH treatment on cardiac histopathological changes post-myocardial infarction. The infiltration of inflammation and fibrosis in left ventricle was visualized by HE stain and Mallory’s trichrome stain, respectively. On the heart 28 day post-myocardial infarction, the infarcted zone became thin, mainly consisted of fibrotic scar tissue and inflammatory cells, few cardiomyocytes could be seen in this area. In the peri-infarcted zone, a large amount fibrotic tissue and inflammatory cells was found inserting into the cardiomyocytes. The remote zone dominantly consisted of cardiomyocytes, interspersed with a few fibrotic tissue and inflammatory cells. As showed in **Figures 3, 4**, treatment of SH alleviated the infiltration of inflammation and fibrosis in all these three zones. Moreover, the inflammatory and fibrotic changes in left ventricle were more suppressed by high dose SH.

### Sodium Houttuyfonate Suppressed the Cardiac Expression of Inflammatory Cytokines and Collagen Post-myocardial Infarction

**FIGURE 5:**
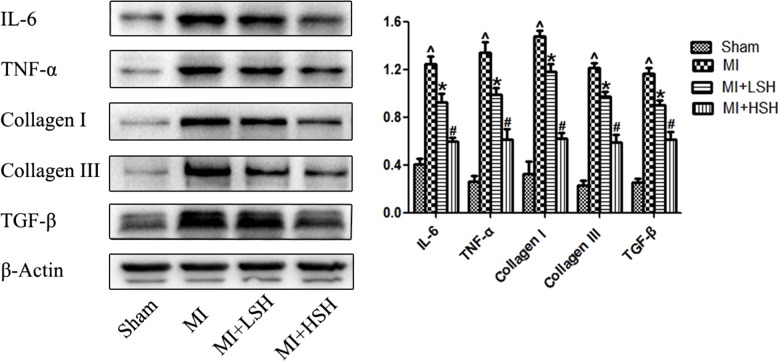
Western blotting revealed the cardiac expressions of cytokines and collagens post-myocardial infarction were reduced by SH treatment. Sham, *n* = 6; MI, *n* = 6; MI+LSH, *n* = 6; MI + HSH, *n* = 6. Data were expressed as means ± SD. ^∧^Compared with Sham group, *p* < 0.05; ^∗^compared with MI group, *p* < 0.05; ^#^compared with MI+LSH group, *p* < 0.05.

To further confirm the effect of SH on cardiac inflammation and fibrosis post-myocardial infarction, the cardiac expressions of TNF-α, IL-6, TGF-β, collagen I and collagen III were investigated by western blotting and compared among the four groups. As shown in **Figure 5**, compared with Sham group, there was obvious elevation of TNF-α, IL-6, TGF-β, collagen I and collagen III in MI group (*p* < 0.05). SH treatment dose-dependently suppressed the expression of TNF-α, IL-6, TGF-β, collagen I and collagen III and (*p* < 0.05), with high dose SH exhibiting a more notable inhibitory effect (*p* < 0.05).

### Sodium Houttuyfonate Treatment Activated AMPK and Suppressed NF-κB p65 Post-myocardial Infarction

**FIGURE 6:**
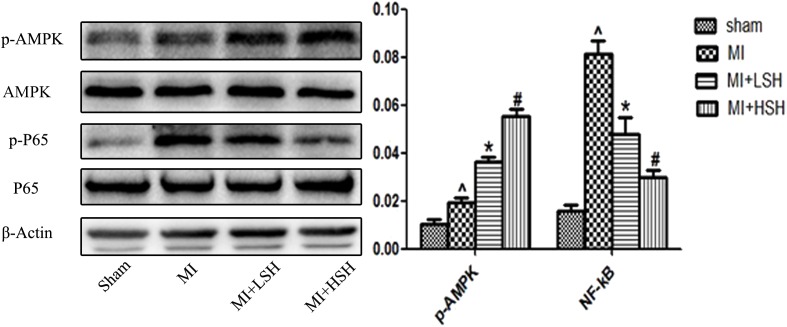
Western blotting revealed that cardiac p-AMPK and p-NF-kB P65 was activated and suppressed by SH treatment, respectively. Sham, *n* = 6; MI, *n* = 6; MI+LSH, *n* = 6; MI+HSH, *n* = 6. Data were expressed as means ± SD. ^∧^Compared with Sham group, *p* < 0.05; ^∗^compared with MI group, *p* < 0.05; ^#^compared with MI+LSH group, *p* < 0.05.

As the AMPK signal pathway was critically involved in the adaptive response to hypoxic and ischemic stress, and *H. cordata* has been reported could elicit and enhance the activation of AMPK in other animal models of diseases, we further investigated whether there is an influence of SH on AMPK during post-infarct remodeling. As shown in the **Figure 6**, compared with Sham group, there was an increased phosphorylation of AMPK in MI group (*p* < 0.05). SH treatment further increased the phosphorylation of AMPK (*p* < 0.05), and a more enhanced phosphorylation of AMPK was observed in the MI+HSH group (*p* < 0.05). The NF-κB signaling pathway, which was a classical pathway of inflammation, was recently demonstrated to promote cardiac remodeling after MI. We also investigated the SH effect on NF-κB signal pathway during post-infarct remodeling. As shown in the **Figure 6**, compared with Sham group, there was an obviously increased phosphorylation of NF-κB p65 in MI group. However, the phosphorylation of NF-κB p65 was obviously inhibited in SH treatment groups (*p* < 0.05), and a much higher inhibitory effect NF-κB p65 was found in MI+HSH group (*p* < 0.05).

### Sodium Houttuyfonate Exerted a Protective Effect on H9C2 Post-hypoxia via Activating AMPK and Suppressing NF-κB Pathway

**FIGURE 7:**
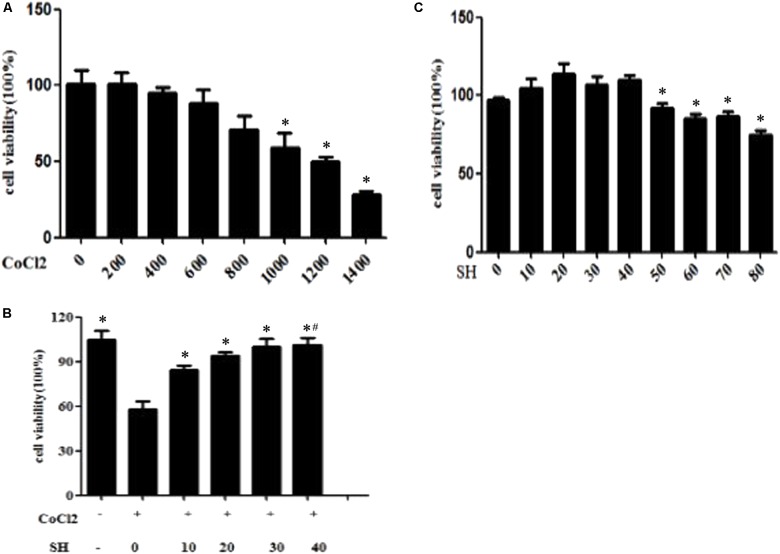
CCK-8 assay showed that SH treatment improved the cell viability of H9C2 post CoCl_2_-induced hypoxia. **(A)** H9C2 were treated with various concentrations of CoCl_2_ (0, 200, 400, 600, 800, 1000, 1200, and 1400 μM, *n* = 6 in each concentration of CoCl_2_) alone for 24 h, the concentration of 1000 μM CoCl_2_ resulted in half cell death. ^∗^Compared with 0 μM CoCl_2_ treatment, *p* < 0.05. **(B)** H9C2 were treated with various concentrations of SH (0, 10, 20, 30, 40, 50, 60, 70, and 80 μg/ml, *n* = 6 in each concentration of SH) alone for 24 h. ^∗^Compared with 0 μg/mL SH treatment, *p* < 0.05. **(C)** The H9C2 induced hypoxia by CoCl_2_ were treated with SH (0, 10, 20, 30, and 40 μg/mL, *n* = 6 in each concentration of SH) for 24 h, respectively. ^∗^Compared with 0 μg/mL SH treatment, *p* < 0.05. ^#^Compared with 30 μg/ml SH treatment, *p* > 0.05.

To further elucidate the mechanism underlying SH treatment, H9C2 were induced hypoxia by CoCl_2_ to simulate myocardial infarction in animal model. As shown in **Figure 7A**, based on CCK-8 assay, the CoCl_2_ reduced cell viability in a concentration-dependent manner and the concentration of 1000 μM resulting in half cell death was applied to all cell experiments in this study. Meanwhile, H9C2 were treated with various concentrations of SH (0–80 μg/ml) alone for 24 h. Cell viability did not change significantly when cells were treated with SH at different concentrations ranging from 10 to 40 μg/ml (*p* > 0.05), whereas SH at concentrations of 50–80 μg/ml induced slight cell viability reduction (*p* < 0.05), as shown in **Figure 7B**. The H9C2 cells induced chemical hypoxia by 1000 μM CoCl_2_ were then treated with SH (0, 10, 20, 30, and 40 μg/ml) for 24 h, respectively. In the range of 0–30 μg/ml SH, CoCl_2_-induced cytotoxicity was inhibited and cell viability was enhanced in a dose-dependent manner (*p* < 0.05), as shown in **Figure 7C**. There was no significant changes in cell viability between administration of 30 μg/ml and 40 μg/ml SH (*p* > 0.05).

**FIGURE 8:**
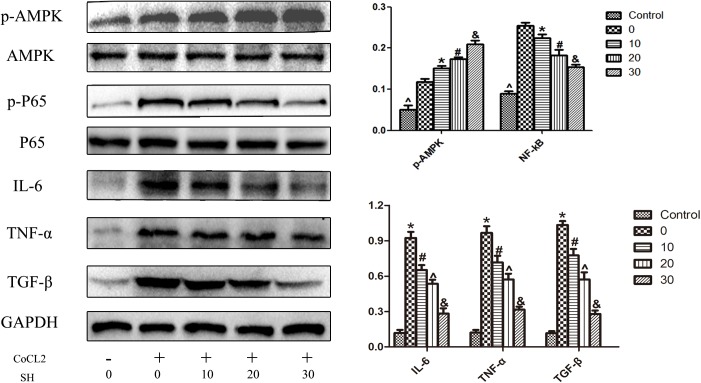
Western blotting showed that SH decreased the expression of inflammatory cytokines, increased the activation of AMPK, suppressed the activation of NF-kB P65 on H9C2 post COCl_2_-induced hypoxia. Control, *n* = 6; 0, CoCl_2_ + 0 μ/ml SH, *n* = 6; 10, CoCl_2_ + 10 μg/ml SH, *n* = 6; 20, CoCl_2_ + 20 μg/ml SH, *n* = 6; 30, CoCl_2_ + 30 μg/ml, *n* = 6. ^∗^Compared with control, *p* < 0.05; ^#^compared with 0 μg/ml SH post CoCl_2_, *p* < 0.05; ^∧^compared with 10 μg/ml SH post CoCl_2_, *p* < 0.05; ^&^compared with 20 μg/ml post CoCl_2_, *p* < 0.05.

**FIGURE 9:**
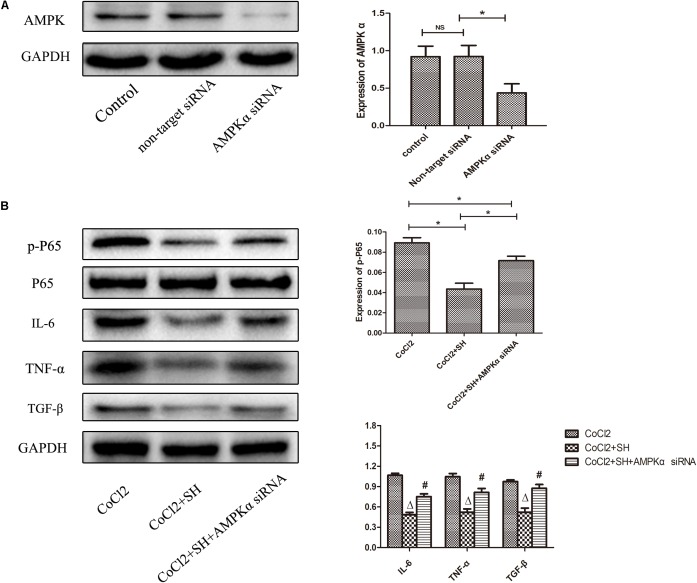
Transfection H9C2 with AMPKα siRNA reversed the suppression of NF-kB p65 and pro-inflammatory cytokines by SH post-hypoxia. **(A)** The expression of AMPK of H9C2 transfected with AMPKα siRNA. Control, *n* = 6; non-target siRNA, *n* = 6; AMPKα siRNA, *n* = 6. NS indicated *p* > 0.05, ^∗^indicated *p* < 0.05. **(B)** The expression of NF-kB p65, IL-6 and TNFα of H9C2 transfected with AMPKα siRNA. CoCl_2_, *n* = 6; CoCl_2_+SH, *n* = 6; CoCl_2_+SH+AMPKα siRNA, *n* = 6. ^∗^Indicated *p* < 0.05; ^Δ^compared with CoCl_2_ group, *p* < 0.05; ^#^compared with CoCl_2_+SH group, *p* < 0.05.

The effect of SH on H9C2 post-hypoxia was further investigated by western blotting. As shown in **Figure 8**, in the range of 0–30 ng/ml, SH decreased the expression of IL-6, TNF-α and TGF-β in a dose-dependent manner (*p* < 0.05). The changes of AMPK and NF-κB signal pathway were also explored. In the range of 0–30 μg/ml SH treatment, with the reduction of inflammatory cytokines, the AMPK and NF-κB p65 were gradually activated and suppressed. Whether there was a correlation between AMPK and NF-κB pathway under the treatment of SH post-hypoxia was further examined. After transfected with AMPKα siRNA, the expression of AMPK in H9C2 was remarkably down-regulated (*p* < 0.05). As shown in **Figure 9**, SH treatment post-hypoxia suppressed p-NF-κB p65 and inflammatory cytokines (IL-6, TNF-α, and TGF-β) in H9C2 (*p* < 0.05). However, this protective effect of SH was reversed by AMPK knockdown. With the knockdown of AMPK, the NF-κB p65 and inflammatory cytokines was significantly up-regulated (*p* < 0.05).

## Discussion

In the present study, the effect of SH on post-infarct remodeling was first and comprehensively investigated. We found that 4-week administration of SH post-myocardial infarction alleviated the adverse cardiac remodeling, ameliorated the inflammatory and fibrotic responses of cardiac tissue. Additionally, as treatment with SH increased AMPK activation and suppressed NF-κB activation in our study, we suspected that the cardioprotective effect of SH post-myocardial infarction may be contributed to it regulating these signal pathways. To further explore the mechanism underlying the benefits of SH treatment, H9C2 were induced hypoxia to simulate myocardial infarction in animal model, we found SH treatment reduced the expression of inflammatory cytokines, increased the activation of AMPK and decreased the activation of NF-κB on H9C2 in a dose-dependent manner. Intriguingly, transfection H9C2 with AMPKα siRNA remarkably blunted the protective effect of SH post-hypoxia. Based on these findings, we concluded that SH treatment post-myocardial infarction could attenuate post-infarct remodeling, alleviate cardiac inflammatory and fibrotic responses, and the underlying mechanism was related to activating AMPK and suppressing NF-κB signal pathway.

During past decades, with the widespread application of anti-remodeling drugs, angiotensin-converting-enzyme inhibitor (ACEI), angiotensin receptor blocker (ARB) and β-blocker, the prognosis of patients with myocardial infarction have been largely improved. Nevertheless, a part patient population post-myocardial infarction still developed into progressive cardiac remodeling and heart failure, due to no response to these anti-remodeling drugs or intolerance to the side effects caused by these agents (Gullo et al., 2014). Therefore, new and efficient anti-remodeling drugs are still urgently needed. Recent studies have revealed that post-infarct remodeling was associated with the persisting low-level inflammatory response and tissue fibrosis, moreover, modulation of the inflammatory and fibrotic responses could, to some extent, reverse the process of post-infarct remodeling (Li et al., 2006; Takahashi et al., 2008; Hamid et al., 2011; Sobirin et al., 2012; Shivshankar et al., 2014; Wang B. et al., 2017). *H. cordata* is a classical Chinese herb used in traditional Chinese medicine for inflammatory diseases, such as pneumonia. Recently, SH, which was a pure compound extracted from *H. cordata*, has been found exerting anti-inflammatory and anti-fibrotic effects on animal and cell models of various diseases (Gao et al., 2010; Du et al., 2012; Kang and Koppula, 2014a, 2015; Xu et al., 2015; Zhu et al., 2015; Wu et al., 2017). On LPS-induced bovine endometrial epithelial cell inflammation, SH treatment significantly decreased the expression of pro-inflammatory cytokines and alleviated the inflammatory process. On chronic obstructive pulmonary disease (COPD) inflammatory model rats induced by combination usage of cigarette smoke and LPS, the pathological changes of lung tissues were considerably alleviated, as well as pro-inflammatory cytokines significantly decreased by SH treatment. SH treatment was also found decreasing TNFα level and attenuating ventricular remodeling induced by abdominal aortic banding in rats. On a rapid pulmonary fibrosis rat model, SH treatment repaired lung injury and reduced pulmonary fibrosis in a dose-dependent manner. As SH has been confirmed a potent anti-inflammatory and anti-fibrotic agent under diseased situations by previous studies, in this study, we aimed to investigate whether SH could exert an anti-remodeling effect post-myocardial infarction by alleviating inflammatory and fibrotic responses. Our study for the first time clarified that SH treatment can alleviate the post-infarct inflammation and fibrosis, reverse post-infarct remodeling and can be a promising drug therapy for chronic heart failure caused by myocardial infarction.

To elucidate the mechanism underlying SH treatment during post-infarct remodeling, the AMPK and NF-κB signal pathways were further investigated. It has been reported that AMPK was one of the downstream signal pathways of Cordata Houttuynia treatment under diseased situations (Kang and Koppula, 2014b; Wang J.H. et al., 2017). On the other hand, AMPK has been widely known involved in coordinating a series pathophysiological responses under ischemic stress, and pharmacological activation of AMPK has been found preventing inflammation and cellular death, reducing cardiac remodeling post-myocardial infarction (Calvert et al., 2008; Peairs et al., 2009; Chiang et al., 2017; Kim et al., 2017; Quan et al., 2018). In our study, we found that SH treatment elevated the activation of AMPK on post-infarct heart and post-hypoxia H9C2. Therefore, it was a reasonable guess that AMPK was critically involved in the anti-remodeling effect of SH post-myocardial infarction.

With the increased activation of AMPK pathway, enhanced suppression of NF-κB pathway was also observed by SH treatment. Moreover, in H9C2 post-hypoxia, knockdown of AMPK by siRNA blunted the suppression of NF-κB and inflammatory cytokines by SH. Based on this observation, we proposed that the cardioprotective effect of SH was related to downregulating NF-κB pathway by upregulating AMPK pathway. The NF-κB signaling pathway, which was widely known as a classical pathway involved in inflammatory and fibrotic responses during various diseases, was recently demonstrated promoting adverse cardiac remodeling post-myocardial infarction by aggravating and prolonging the inflammatory and fibrotic processes (Martinez-Martinez et al., 2017; Wang B. et al., 2017). There were emerging evidences indicating AMPK signaling can inhibit the pathological responses induced by the nuclear factor-κB (NF-κB) system. A plethora of studies revealed that AMPK did not suppress NF-κB signaling directly, its inhibition of NF-κB was realized indirectly via its downstream mediators, e.g., Sirtuin-1 (SIRT1), Forkhead box O (F oxO) family, and peroxisome proliferator-activated receptor γ co-activator 1α (PGC-1α) (Salminen et al., 2011). Based on these investigations, we proposed that SH treatment attenuated post-infarct remodeling and exerted cardioprotective effect via activating AMPK and suppressing NF-κB pathway.

In our study, by knockdown AMPK in H9C2, we confirmed the AMPK signal regulating NF-κB pathway during SH treatment under hypoxia. However, this relationship between AMPK and NF-κB pathway under SH treatment was not further investigated *in vivo*. We once considered adopting AMPKα knockout animal to testify the effect of SH on post-infarct remodeling. Whereas, it has been demonstrated that knockout of AMPK subunits in animal could result in cardiomyopathy and cardiac remodeling, which would complicate our investigation of SH on post-infarct remodeling (Dolinsky and Dyck, 2006; Sung et al., 2015). In addition, though the effect of SH treatment post-hypoxia was only tested on cardiomyoblast H9C2 but not on cardiac fibroblast, it still can give a reasonable explanation of SH’s anti-inflammatory and anti-fibrotic potent observed on myocardial infarcted rat. We have confirmed the SH reduced the proinflammatory cytokines (IL-6 and TNF-α) on H9C2 post-hypoxia. Meanwhile, we also found that SH treatment lead to a significant reduction of TGF-β. TGF-β has been already confirmed a central cytokine of fibrogenesis and play an essential role in the pathogenesis of fibrosis. A large amount studies have reported that after myocardial infarction, TGF-β was markedly induced and rapidly activated in the infarcted myocardium (Dobaczewski et al., 2011). Upon TGF-β stimulation, pathways related to tissue fibrosis were activated and collagen deposition was initiated during cardiac remodeling. With the reduction of TGF-β by SH observed on H9C2 post-hypoxia, the anti-fibrotic effect of SH could be better understood.

## Conclusion

In this study, we found SH treatment could alleviate post-infarct inflammatory and fibrotic responses, attenuate post-infarct remodeling in a dose-dependent manner. The underlying mechanism was associated with activation of AMPK and suppression of NF-κB pathway.

## Author Contributions

CZ and X-WL designed and drafted the original research. CZ, J-FL, Z-HL, ST, W-QL, Y-ZL, HL, Z-RL, and J-HC performed the experiments. All authors approved it for publication.

## Conflict of Interest Statement

The authors declare that the research was conducted in the absence of any commercial or financial relationships that could be construed as a potential conflict of interest.
